# Naturally Occurring Autoantibodies against Tau Protein Are Reduced in Parkinson's Disease Dementia

**DOI:** 10.1371/journal.pone.0164953

**Published:** 2016-11-01

**Authors:** Yannick Kronimus, Alexandra Albus, Monika Balzer-Geldsetzer, Sarah Straub, Elisa Semler, Markus Otto, Jens Klotsche, Richard Dodel, LANDSCAPE Consortium, David Mengel

**Affiliations:** 1 Department of Neurology, Philipps-University, Marburg, Germany; 2 Department of Neuro-geriatrics, University Clinic, Essen, Germany; 3 Department of Neurology, University of Ulm, Ulm, Germany; 4 German Rheumatism Research Centre Berlin, a Leibniz Institute, Berlin, Germany; 5 Charité University Medicine Berlin, Institute for Social Medicine, Epidemiology and Health Economics, Berlin, Germany; Hertie Institute for Clinical Brain Research and German Center for Neurodegenerative Diseases, GERMANY

## Abstract

**Background and Objective:**

Altered levels of naturally occurring autoantibodies (nAbs) against disease-associated neuronal proteins have been reported for neurodegenerative diseases, such as Alzheimer's (AD) and Parkinson's disease (PD). Recent histopathologic studies suggest a contribution of both Lewy body- and AD-related pathology to Parkinson's disease dementia (PDD). Therefore, we explored nAbs against alpha-synuclein (αS), tau and β-amyloid (Aβ) in PDD compared to cognitively normal PD patients.

**Materials and Methods:**

We established three different ELISAs to quantify the nAbs-tau, nAbs-αS, and nAbs-Aβ levels and avidity towards their specific antigen in serum samples of 18 non-demented (PDND) and 18 demented PD patients (PDD), which were taken from an ongoing multi-center cohort study (DEMPARK/LANDSCAPE).

**Results:**

PDD patients had significantly decreased nAbs-tau serum levels compared to PDND patients (*p* = 0.007), whereas the serum titers of nAbs-αS and nAbs-Aβ were unchanged. For all three nAbs, no significant differences in avidity were found between PDD and PDND cohorts. However, within both patient groups, nAbs-tau showed lowest avidity to their antigen, followed by nAbs-αS, and nAbs-Aβ. Though, due to a high interassay coefficient of variability and the exclusion of many samples below the limit of detection, conclusions for nAbs-Aβ are only conditionally possible.

**Conclusion:**

We detected a significantly decreased nAbs-tau serum level in PDD patients, indicating a potential linkage between nAbs-tau serum titer and cognitive deficits in PD. Thus, further investigation in larger samples is justified to confirm our findings.

## Introduction

Altered levels of naturally occurring autoantibodies (nAbs) against disease-associated proteins have been reported for neurodegenerative diseases, such as Alzheimer's (AD) und Parkinson's disease (PD) [1–6]. Although the source and function of these autoantibodies is not understood thus far, evidence has accumulated that nAbs are involved in maintaining physiologic homeostasis. They mostly belong to class G or M immunoglobulins and recognize and induce clearance of altered self-structures, including oxidatively damaged components, dying cells and aggregated or misfolded proteins [7–10]. Therefore, altered properties or levels of nAbs against specific proteins may be a disease-causing factor.

Dementia is a frequent and disabling symptom in PD. However, the pathological cause of Parkinson's disease dementia (PDD) remains unclear [11]. PD is characterized by the loss of dopaminergic neurons in the substantia nigra along with the presence of Lewy bodies and Lewy neurites in surviving neurons [12]. A core content of these intraneuronal inclusions is α-synuclein (αS). In advanced disease stages, Lewy body pathology can also diffusely affect limbic and neocortical brain regions [13,14]. Clinically, pervasion of Lewy body pathology to these brain areas has been linked to cognitive deterioration, which can progress to dementia in PD patients [15]. In addition to cortical Lewy body pathology, AD-type pathology has been described as a neuropathological substrate of PDD [16]. AD-type pathology includes both extraneuronal deposition of β-amyloid (Aβ) and intraneuronal accumulation of hyperphosphorylated tau protein.

In animal studies, β-amyloid has been shown to accelerate α-synuclein deposition, suggesting a synergistic interaction between the described proteins in PD pathology, particularly in PDD [17].

NAbs recognizing the three described proteins, including nAbs-αS, nAbs-tau, and nAbs-Aβ, have been detected in human serum samples. Interestingly, the serum levels of nAbs-αS, nAbs-tau, and nAbs-Aβ differed significantly between patients and controls in neurodegenerative diseases [1,2,4,5]. These findings prompted us to perform an explorative study to investigate changes in the nAbs levels in PDD compared to PD patients to identify a potential diagnostic biomarker for PDD.

## Materials and Methods

### Patients

To implement the explorative study, samples and clinical as well as neuropsychological data of eighteen non-demented PD (PDND) patients and eighteen PDD patients were taken from the DEMPARK/LANDSCAPE cohort study [18]. Clinical characterization of our study cohort is shown in Table 1. Patients were allocated into both groups following matching for age (± 5 years) and gender. In addition, we only included patients with disease duration > 5 years, as disease duration is a major risk factor for dementia in PD. Successful matching is outlined in Table 1. For enrolment in the DEMPARK/LANDSCAPE study, participants had to meet criteria for the diagnosis ‘idiopathic PD’ according to the UK Parkinson's Disease Society Brain Bank clinical diagnostic criteria [19]. A diagnosis of PDD was made according to Level II Movement disorder society task force criteria [20]. The study was conducted in accordance with the declaration of Helsinki [21]. The study protocol was approved by the Ethics Committee of the Philipps-University Marburg (No.: 178/07) and subsequently by the Ethics Committees of the associated centers, which are part of the Dempark/Landscape study. Separate consent was required for each module. Patients were only included after giving their written informed consent. In the case that a patient suffered from dementia the legal guardian gave written informed consent.

**Table 1:** Patient characteristics.

	PDND	PDD	*p*-value^a^^)^	PD	bvFTD	*p*-value^b^^)^
No. of patients	18	18		36	20	
Male/female	13/5	13/5		26/10	12/8	
Age at taking blood sample [mean ± SD]	71.6 ± 4.1	71.9 ± 4.7	0.851	71.8 ± 4.3	61.7 ± 11.3	0.001
Age range	64–78	61–79		61–79	38–82	
Disease duration [mean ± SD]^c^^)^	9.6 ± 3.1	11.6 ± 4.8	0.226	10.6 ± 4.1	3.1 ± 2.8	< 0.001
Age at onset of disease [mean ± SD]^d^^)^	62.0 ± 5.4	60.3 ± 7.2	0.425	61.1 ± 6.4	58.7 ± 11.2	0.370
H&Y stage, median [min–max]	2.0 [1–4]	3.0 [2–5]	< 0.001	Not determined, because H&Y stage was not examined for FTD		
MMSE	28.7 ± 1.1	25.9 ± 2.7	< 0.001	27.3 ± 2.4	24.9 ± 4.5	0.008

Abbreviations: PDND = non-demented Parkinson's disease patients; PDD = Parkinson's disease with dementia; PD = patients with Parkinson's disease (PDND and PDD patients combined); bvFTD = patients with behavioral variant of frontotemporal dementia; H&Y = Hoehn & Yahr; MMSE = Mini Mental State Examination. For statistical analysis, the Student's *t*-test and Mann-Whitney-U test were applied.

^a^ PDND compared to PDD

^b^ PD (PDND and PDD patients combined) compared to bvFTD

^c^ defined as years since initial diagnosis for PD and years since first symptoms for bvFTD

^d^ defined as age at initial diagnosis for PD and age at first symptoms for bvFTD

As an additional group we included patients with the behavioral variant of frontotemporal dementia (bvFTD) into our analysis. Patients were diagnosed according to recent Rascovsky criteria [22]. and were recruited at the department of Neurology in Ulm. Collection and analysis of samples were approved by the Ethics Committees in Ulm (approval number 39/11). All patients or their next relatives in case of severe dementia gave written informed consent to their participation in the study. The clinical and demographic characteristics are outlined in Table 1.

### ELISA

#### NAbs-tau-ELISA

Tau-441 (rPeptide, Bogart, GA, USA) was dissolved in ultrapure water, stored at -80°C (up to 8 weeks) and treated in an ultrasonic bath for 15 min before coating. Sixty wells of high-binding 96-well ELISA plates (Immulon Microtiter Plates 2HB “U” Bottom, Thermo Scientific, Braunschweig, Germany) were coated with 50 μl of 5 μg/ml recombinant tau-441 peptide in 0.1 M sodium phosphate buffer (2.62 g/l sodium dihydrogen phosphate monohydrate, 21.71 g/l disodium hydrogen phosphate heptahydrate in ultrapure water, pH 7.4). The 36 remaining wells were incubated with sodium phosphate buffer only (uncoated wells). After 24 h at 4°C, the wells were blocked with 180 μl blocking buffer (1x Roti®Block + 0.1% Tween-20, Carl Roth, Karlsruhe, Germany) for additional 24 h at 4°C. Serum samples were diluted 1:50 in blocking buffer (1x Roti®Block + 0.1% Tween-20) and added to coated and uncoated wells as double triplets (50 μl/well). A standard curve (including blank) was generated by sequential dilution of a monoclonal Anti-tau antibody (Anti-tau antibody (ab22261), Abcam, Cambridge, MA, USA) in blocking buffer. In addition, a pooled serum standard, generated by mixing a variety of patient samples, was included in every assay. Plates were incubated with samples for 1 h at ambient temperature (AT) and shaken on an orbital platform shaker (Unimax 1010, Heidolph, Schwabach, Germany) at 100 rpm.

To evaluate the avidity of nAbs-tau, 8 M of urea per serum sample was added to one of the triplets of both coated and uncoated wells for 60 s as described by Fialova et al. [23]. Simultaneously, washing solution was added to the remaining triplets and samples for standard curve. The quotient of serum samples treated with urea, divided by the serum samples treated with washing solution, was set as the avidity because the denaturing agent urea breaks down the antibody-antigen complexes. Correspondingly, avidity reduction was calculated via avidity subtracted from 1. Afterwards, a second blocking step with 180 μl of blocking buffer was performed, and the sample was shaken with 100 rpm for 1 h at AT. Detection antibodies were added to the wells for 1 h at AT and were shaken at 100 rpm (50 μl/well; for standard curve: biotinylated Goat Anti-Mouse IgG Antibody, Vector, Burlingame, CA, USA, 1:20,000 in blocking buffer; for serum samples and standard: Goat Anti-Human IgG, Fc-Biotin, Dianova, Hamburg, Germany, 1:20,000 in blocking buffer). Then, the samples were incubated with 50 μl/well of streptavidin-peroxidase (Streptavidin-HRP, R&D Systems, Minneapolis, MN, USA, 1:200 in blocking buffer) and shaken at 100 rpm for 20 min at AT. The assay was developed using 50 μl/well of 3,3‘,5,5‘-tetramethylbenzidine (TMB, Merck, Darmstadt, Germany). After 20 min, the reaction was stopped by adding 25 μl/well of 5% sulfuric acid (Carl Roth, Karlsruhe, Germany). The optical density (OD) of the yellow color was measured at 450 nm using a microplate reader (Infinite 200 PRO Microplate Reader, Tecan, Crailsheim, Germany). All incubation steps were performed in the dark. Up to and including TMB addition, the wells were washed three times with 300 μl 1x PBS (8 g/l sodium chloride, 0.2 g/l potassium chloride, 1.44 g/l disodium hydrogen phosphate, and 0.24 g/l potassium dihydrogen phosphate) + 0.05% Tween-20 after each step using an Amersham Biotrak II Plate Washer (GE Healthcare Europe, Freiburg, Germany).

#### NAbs-αS-ELISA

Only differences compared to the nAbs-tau ELISA are outlined. Recombinant αS (Alpha-Synuclein, rPeptide, Bogart, USA) was dissolved in ultrapure water and stored at -20°C (up to 8 weeks). Coating was performed with 3 μg/ml αS in PBS (Biochrom GmbH, Berlin, Germany). Uncoated wells were incubated with PBS. A standard curve was generated with nAbs-αS, which were isolated from human intravenous immunoglobulin G (IVIg Gamunex, Grifols GmbH, Frankfurt, Germany) via affinity chromatography as previously described [1]. Goat Anti-Human IgG, Fc-Biotin (Dianova GmbH, Hamburg, Germany, 1:20,000 in blocking buffer) was used as the detection antibody.

#### nAbs-Aβ-ELISA

Only differences compared to the nAbs-tau ELISA are outlined. Recombinant Aβ_1–42_ (Amyloid-Beta 1–42, rPeptide, Bogart, USA) was dissolved in 10% DMSO, subsequently diluted in 90% 0.05 M sodium carbonate buffer (1.59 g/l sodium carbonate, 2.93 g/l sodium hydrogen carbonate in ultrapure water, pH 9.6), treated in an ultrasonic bath for 10 min and stored at -80°C (up to 4 weeks). Wells were coated with 10 μg/ml Aβ. A standard curve was generated with nAbs-Aβ, which were isolated from human intravenous immunoglobulin G (IVIg Gamunex, Grifols, Frankfurt, Germany) via affinity chromatography [24]. Serum samples were diluted 1:25 in blocking buffer and goat Anti-Human IgG, Fc-Biotin (Dianova GmbH, Hamburg, Germany, 1:20,000 in blocking buffer) was used as the detection antibody.

### Analysis

First, the mean OD of all triplets was determined. To eliminate background signals, the blank was subtracted from each standard and serum signals of uncoated wells were subtracted from the corresponding serum signals of coated wells. With known concentrations and ODs of the standards, a standard curve for all ELISA plates was generated using the 4 parameter logistic nonlinear regression model. The quotient of the serum sample treated with urea divided by the serum sample treated with washing solution was calculated to generate the avidity reduction. To check the interassay coefficient of variability, all ELISA plates contained a pooled serum standard. If the nAbs levels were not evaluable due to one of the following criteria, the corresponding values were restricted from further analysis.

1. background signal higher than sample signal
2. sample signal lower than limit of detection, calculated as
  LOD = (mean_blank_ + 1.645(SD_blank_)) + 1.645(SD_lowest sample_) [25]
3. increasing signal after urea treatment

Therefore, the evaluation of the nAbs-tau-ELISA included 14 PDND (11 with urea) and 15 PDD (12 with urea) samples, the nAbs-αS-ELISA 17 PDND (14 with urea) and 17 PDD (also 17 with urea) patients, respectively. After application of the criteria, 13 PDND (8 with urea) and 11 PDD (7 with urea) samples were incorporated in the evaluation of the nAbs-Aβ-ELISA.

In an additional analysis, we also measured nAbs titers of bvFTD patients. Here, the evaluation of the nAbs-tau-ELISA included 18, the nAbs-αS- and nAbs-Aβ-ELISA 20 bvFTD patients, respectively.

### Statistical Analysis

For statistical analysis, SPSS (version 22.0.0.0, IBM SPSS Statistics, Chicago, USA) and STATA 12.1 (Stata Statistical Software: Release 12. College Station, TX: StataCorp LP.) was used. Normal distribution of variables of interest was tested by a Kolmogorov-Smirnov test. Group differences between PDND and PDD were tested by generalized linear mixed models to account for the matched case-control study design. Differences within the patient groups were tested using the paired-samples *t*-test or the Wilcoxon signed-rank test. ANOVA or Kruskal-Wallis test with a following post-hoc multiple comparison procedure (Scheffe’s procedure) was used to compare more than two groups. Student's *t*-test or the Mann-Whitney-U test was used for the test of group differences between PD and bvFTD as appropriate. The significance threshold was set to *p < 0.05 (**p < 0.01; ***p < 0.001).

## Results

### High reproducibility of nAbs-tau-ELISA and nAbs-αS-ELISA

**Fig 1:**
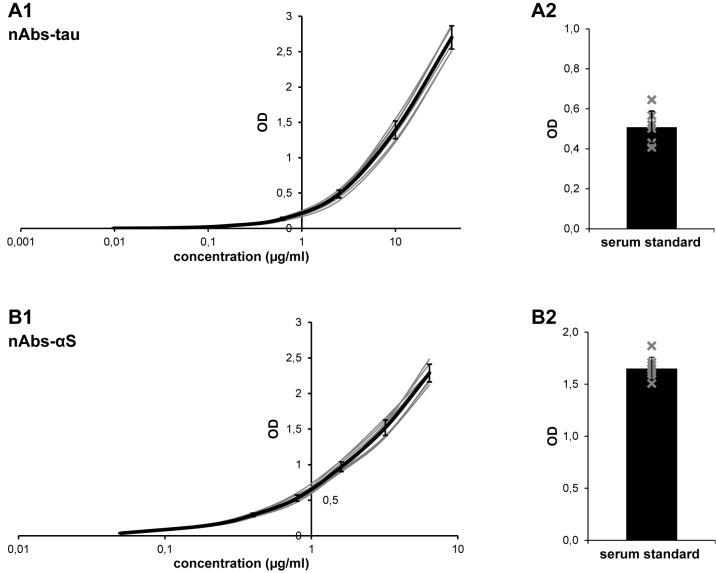
Interassay variance. Figure shows standard curves (A1, B1) of single ELISA plates (grey) and mean standard curves (black) of nAbs-tau-ELISA (A1) and nAbs-αS-ELISA (B1). Furthermore, single (grey crosses) and mean ODs of the pooled serum standard (A2, B2) are displayed for nAbs-tau-ELISA (A2) and nAbs-αS-ELISA (B). Data are shown as the mean ± SD.

First, we tested the interassay coefficient of variability (CV) of our ELISAs using the serum standard from each ELISA plate. The optical density (OD) of the pooled serum standard showed only small differences between all assay plates in nAbs-tau-ELISA (serum standard OD: 0.51 ± 0.08; Fig 1A2) and nAbs-αS-ELISA (serum standard OD: 1.65 ± 0.11; Fig 1B2). The interassay CV was calculated as the percentage of the standard deviation (SD) from the mean value (% CV = SD x 100 / mean). Interassay CV for both the nAbs-tau-ELISA (15.9%; Fig 1A2) and nAbs-αS-ELISA (6.7%; Fig 1B2) was acceptable. In addition, interassay variance of the standard curves of the nAbs-tau-ELISA and the nAbs-αS-ELISA was small, as shown by highly overlapping standard curves (Fig 1A1 and 1B1). In contrast, ODs of the serum standard showed significant variations in the nAbs-Aβ-ELISA, resulting in an interassay CV of 41.7%. Conclusions from the nAbs-Aβ-ELISA are therefore only conditionally possible and are presented in S1 Fig.

We also calculated the intraassay CV as the percentage of SD from the mean for each serum sample. The intraassay CV was 5.5 ± 1.9% for nAbs-tau, 4.9 ± 1.5% for nAbs-αS, and 7.1 ± 3.5% for nAbs-Aβ ELISA (data not shown).

### Reduced nAbs-tau serum level in PDD patients

Serum was obtained from 18 PDND and 18 PDD patients who were between 61 and 79 years of age. The demographic characteristics and clinical features of the groups are outlined in Table 1.

**Fig 2:**
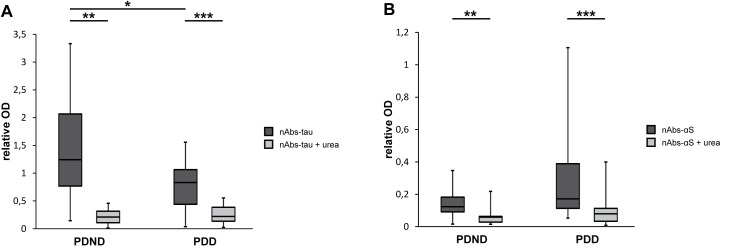
**Distribution of relative serum sample ODs of nAbs-tau- (A) and nAbs-αS-ELISA (B).** The relative serum sample OD (related to serum standard OD) of each non-demented (PDND) and demented Parkinson's disease (PDD) patient was determined without (nAbs) and with urea treatment (nAbs + urea). For an overview of their distribution, box plots show the median, 25% and 75% quartile. 50% of the generated data are located in the box and whiskers represent the minimum and maximum value. NAbs-tau OD showed significant differences between PDND and PDD patients (A; **p = 0.007). Treatment with 8 M urea caused significant reduction of ODs of nAbs-tau (A; PDND: ***p = 0.002; PDD: ***p < 0.001) and nAbs-αS (B; PDND: *p = 0.001; PDD: **p < 0.001).

To compare the nAbs serum levels between PDND and PDD patients, the relative ODs (patient serum sample OD divided by serum standard OD) of serum nAbs are given. The mean relative OD of nAbs-tau was 1.46 ± 0.93 for PDND and 0.75 ± 0.47 for PDD patients. Here, significant differences between both groups were detected (Fig 2A and S1 Table). In contrast, nAbs-αS (PDND: OD = 0.15 ± 0.10, PDD: OD = 0.28 ± 0.28; Fig 2B and S1 Table) showed no significant differences between the groups in terms of the mean relative OD.

In both cases, treatment with 8 M urea resulted in a significant reduction of the mean relative OD (Fig 2A and 2B and S1 Table; nAbs-tau: PDND: OD = 0.22 ± 0.16, PDD: OD = 0.26 ± 0.18; nAbs-αS: PDND: OD = 0.06 ± 0.05, PDD: OD = 0.10 ± 0.11).

In order to provide a more comprehensive picture on nAbs in neurodegenerative diseases, we expanded our investigations to another disorder. We measured nAbs titers in serum of patients suffering from the behavioral variant of fronto-temporal dementia (bvFTD). NAbs-tau and nAbs-αS showed a relative mean OD of 1.63 ± 1.46 and 0.48 ± 0.19, respectively. Significant differences were found for nAbs-αS but not for nAbs-tau when comparing the FTD with the PD group (including PDND and PDD patients). In conclusion, FTD patients exhibited a higher nAbs-αS serum level than patients with Parkinson’s disease (S2A and S2B Fig and S2 Table).

### Unchanged avidity of investigated nAbs between PDND und PDD patients

**Fig 3:**
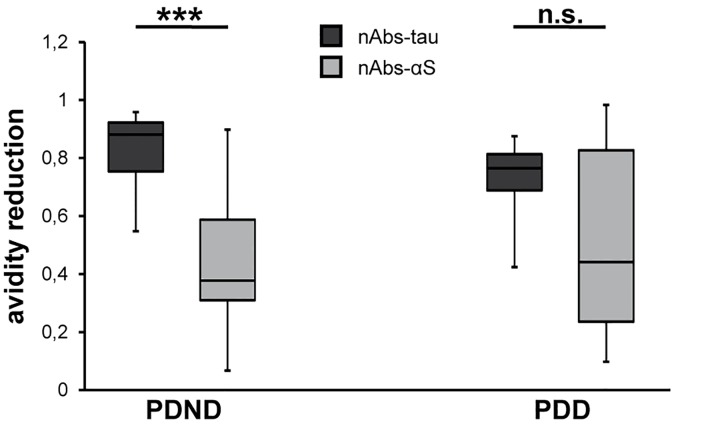
Distribution of the urea-mediated avidity reduction. For each non-demented (PDND) and demented Parkinson's disease (PDD) patient, nAbs-tau and nAbs-αS avidity reduction was calculated. Box plots show the median, 25% and 75% quartile. 50% of the generated data are located in the box and whiskers represent the minimum and maximum value. Significant differences were found within (PDND: ***p < 0.001; PDD: *p* = 0.057) but not between (PDND and PDD: *p* > 0.05) the patient groups.

Next, we explored the avidity reduction of the nAbs to their antigen using treatment with 8 M urea. The decrease in avidity was stronger for nAbs-tau (PDND: OD = 0.82 ± 0.14, PDD: OD = 0.73 ± 0.13) than for nAbs-αS (PD: OD = 0.44 ± 0.26, PDD: OD = 0.52 ± 0.32) (Fig 3 and S3 Table). We tested for statistically relevant differences in the avidity of the nAbs, whereat no one was found between the two patient groups (*p* > 0.05; Fig 3 and S3 Table). However, within the patient groups, especially within the PDND group, we observed differences between the nAbs, indicating the following order in avidity: nAbs-αS > nAbs-tau (PDND: *p* < 0.001, PDD: *p* = 0.057; Fig 3 and S3 Table).

In addition to the OD dependent evaluation, we also analyzed nAbs levels and avidity based on the concentration, which was calculated with the aid of the standard curves and normalized to the PDND group. This second evaluation was run due to a nonexistent proportionality between OD and concentration when using the 4 parameter logistic nonlinear regression model for curve fitting. The results are shown in the supplements but yielded comparable findings (S1C2, S1D2 and S3 Figs and S3 and S4 Tables).

## Discussion

The main finding of the study is a decreased serum level of nAbs-tau in PD patients with dementia compared to non-demented PD subjects. To the best of our knowledge, no study investigating nAbs-tau in PDD subjects has previously been reported. In contrast, the level of nAbs-αS was unchanged between the two study subgroups. Our findings are consistent with previous results from a study by Maetzler et al., who also did not observe differences in the serum level of nAbs-αS between PDND and PDD patients [26]. In addition, we identified nAbs-tau as less avid than the other investigated nAbs.

One reason for a reduction of free serum nAbs-tau in PDD could be the elevated level of the serum tau protein in PDD patients compared to PDND patients, resulting in increased antigen-antibody binding and, thereby, decreasing the free nAbs-tau levels in serum. Increased tau and phospho-tau serum concentrations would be consistent with advanced stages of neurodegeneration and tau pathology–indicated by neurofibrillary tangles–in the brains of PDD compared to PDND patients [11,27–29]. In line with that, increased tau and phospho-tau levels in CSF and blood plasma have been demonstrated in the course of cognitive deterioration in AD [30]. Furthermore, CSF tau and phospho-tau can be increased in PDD compared to PDND patients [31–34]. However, data on tau levels in the blood of PD patients are currently not available due to the fact that, in general, blood titers of tau protein are very low and below the detection level of conventional immunoassays. New ultra-sensitive techniques for the measurement of tau in human blood are currently evaluated by several groups [30]. However, as soon as these techniques become available, a longitudinal study in PD involving the measurement of both nAbs-tau and tau protein levels will help to elucidate the reasons for changes in nAbs levels in the disease process.

In addition to a reduction of free nAbs-tau levels due to increased antigen levels in PDD, another explanation, namely a reverse effect, is possible. Autoantibodies have been proposed to participate in the physiological maintenance of homeostasis of their antigens [35]. Therefore, preexisting reduced levels of nAbs-tau could increase the risk of the accumulation of tau in PD and thus have etiological importance for PDD. In line with that, passive immunization with anti-tau monoclonal antibodies in mouse models ameliorated memory and functional deficits and led to a reduction in the tau-induced histopathological findings [36,37]. Therefore, therapeutic considerations may be based on our findings.

Previous research on nAbs-tau with respect to PD is limited to only one study. Terryberry and colleagues were not able to detect nAbs-tau in serum samples of PD patients [38], but their study suffers from a major drawback as the researchers used bovine instead of human tau protein for their assays. The immunoreactivity between both protein species shows considerable differences, which renders the results difficult to interpret [39]. However, two studies investigating nAbs-tau in AD have been reported. Rosenmann et al. detected nAbs-tau in sera of AD patients as well as healthy subjects, but reported unchanged levels between both groups [5]. In a study by Bartos et al., serum nAbs-tau did also not differ between AD patients and several control groups (cognitively normal elderly controls, a mixed group of other dementias, and patients suffering from inflammatory disorders) [40]. In light of these results, our data suggest a disease-specific role of nAbs-tau in PD, especially for the development of cognitive impairments during the disease.

In order to investigate autoantibodies in another neurodegenerative disease we also determined their serum levels in patients suffering from bvFTD. FTD is a histopathologically heterogeneous disorder comprising tau, TAR DNA-binding protein-43, and fused in sarcoma protein pathology [41]. This is the first report on nAbs in bvFTD. We found comparable levels of nAbs-tau between PD and bvFTD patients. Interestingly, nAbs-αS serum levels were significantly increased in bvFTD patients compared to PD patients. This finding is consistent with previous results of our group that demonstrated reduced nAbs-αS in PD patients compared to healthy subjects indicating a disease-specific role of nAbs-αS in synucleinopathies such as PD [1].

The functional binding capacity of nAbs depends on both the concentration and avidity towards their respective antigen. In line with that, an etiological importance of an altered antibody avidity has been reported for various diseases, e.g. tuberculosis [42] and autoimmune disorders [43]. We tested the avidity of nAbs in serum of PDND and PDD patients, but found no significant differences between both groups. However, within both patient groups, nAbs-tau showed lowest avidity to their antigen. In contrast, studies reporting on multiple sclerosis as well as other neurological diseases discovered anti-tau antibodies in patients and controls in both human CSF and serum with distinct higher avidity [23,44]. The relevance of our finding remains unclear thus far. However, differences in the avidity of the investigated nAbs towards their neuronal antigen could indicate diversity in the autoantibody pool so as there may exist different subgroups of nAbs with specific binding characteristics. Our findings warrant further investigation to better characterize nAbs in terms of their antigen specificity and avidity.

Despite a careful study design, our assays had certain limitations. The nAbs-Aβ-ELISA showed a high interassay variability. Aggregation of Aβ during the coating process is a possible explanation for this effect [45]. In contrast to our assay design, many ELISAs that investigate nAbs-Aβ antibody titers and that have been reported by others were not controlled for typical quality criteria like unspecific background, interassay variability, or limit of detection and thus their assay performance remains unknown.

The avidity of serum nAbs-tau, nAbs-αS, and nAbs-Aβ towards their antigen differed but was unchanged between the two study subgroups, PDD and PDND patients. However, our method using urea as a denaturation agent to break down antibody-antigen binding has limitations. A more sophisticated technique, such as surface plasmon resonance analysis, would provide more useful information in terms of the binding kinetics and strength.

Due to the exploratory character of this study, our result of an altered nAbs-tau serum level in PDD patients needs to be replicated in a larger study. Furthermore, longitudinal evaluation of nAbs levels and their correlation with both the specific antigen concentration and repetitive neuropsychological cognitive assessments at different time points would be of particular interest.

## Supporting Information

S1 Fig Results of the nAbs-Aβ-ELISA. **A+B.** Considerable variation in the standard curves (A; grey: standard curves of single ELISA plates, black: mean standard curve) and a high interassay coefficient of variability (B; 41.7%; grey crosses: serum standard ODs of single ELISA plates, black: mean serum standard OD) demonstrate a low reproducibility. **C:** The relative serum sample OD (related to serum standard OD) of each non-demented (PDND) and demented Parkinson's disease (PDD) patient was determined without (nAbs) and with urea treatment (nAbs + urea). Based thereon and with the aid of the standard curves, nAbs-Aβ concentrations (normalized to the PDND group) were determined. **D:** With both evaluation methods, the urea mediated avidity reduction was determined. Both evaluation strategies are very similar and demonstrate no significant differences between PDND and PDD patients. However, within both patient groups, nAbs-Aβ are most avid, followed by nAbs-αS and nAbs-tau. Box plots show the median, 25% and 75% quartile. 50% of the generated data are located in the box and whiskers represent the minimum and maximum value. Corresponding mean values, standard deviations and *p*-values are outlined in S1, S3 and S4 Tables.
(TIF)

S2 Fig Comparison of nAbs serum titers including patients with behavioral variant of frontotemporal dementia (bvFTD). NAbs-tau- (A), nAbs-αS- (B) and nAbs-Aβ-ELISAs (C) were analyzed using the relative serum sample ODs (related to serum standard OD) of each Parkinson’s disease patient (PD, including PDND and PDD patients) as well as bvFTD patient. For an overview of the distribution of the serum sample ODs, box plots show the median, 25% and 75% quartile. 50% of the generated data are located in the box and whiskers represent the minimum and maximum value. FTD patients showed significantly increased nAbs-αS serum levels compared to PD patients (B; ***p < 0.001).
(TIF)

S3 Fig Evaluation of nAbs-tau- and nAbs-αS-ELISA based on the normalized concentration. Using standard curves, the nAbs-tau and nAbs-αS serum concentration of each non demented (PDND) and demented Parkinson's disease (PDD) patient was determined. After normalization to the PDND group, nAbs-tau (A) and nAbs-αS (B) concentrations as well as the urea mediated avidity reductions (C) were compared between and within the two patient groups. Box plots show the median, 25% and 75% quartile. 50% of the generated data are located in the box and whiskers represent the minimum and maximum value. Corresponding mean values, standard deviations and *p*-values are presented in S3 and S4 Tables.
(TIF)

S1 Table Mean relative optical densities (ODs) of serum samples. Relative serum sample ODs of non-demented (PDND) and demented Parkinson's disease patients (PDD) are shown as the mean ± SD for each ELISA. For avidity determination, the patient serum samples were untreated (OD) or treated with urea (OD + urea). *P*-values: ^a)^ PDND compared to PDD ^b)^PDND: nAbs OD compared to nAbs OD + urea and ^c)^ PDD: nAbs OD compared to nAbs OD + urea.
(PDF)

S2 Table Mean relative optical densities (ODs) of serum samples including patients with behavioral variant of frontotemporal dementia (bvFTD). Relative serum sample ODs of each Parkinson’s disease patient (PD, including PDND and PDD patients) as well as bvFTD patient are shown as the mean ± SD for each ELISA. For statistical analysis, Student's *t*-test or the Mann-Whitney-U test was applied.
(PDF)

S3 Table Avidity reductions. Avidity reductions of the nAbs were calculated for each non-demented (PDND) and demented Parkinson's disease (PDD) patient as the quotient of the urea treated sample divided by the untreated sample. Therefore, the optical density (OD) as well as the concentration based evaluation method was used. Data are shown as the mean ± SD. *P*-values. ^a)^ PDND compared to PDD. ^b)^ nAbs-tau compared to nAbs-αS within patient groups (comparing two groups). ^c)^ nAbs-tau compared to nAbs-αS within patient groups (comparing three groups). ^d)^ nAbs-tau compared to nAbs-Aβ within patient groups (comparing three groups). ^e)^ nAbs-αS compared to nAbs-Aβ within patient groups (comparing three groups).
(PDF)

S4 Table Normalized concentrations (Conc.) of serum samples. Using standard curves, nAbs serum concentrations of non-demented (PDND) and demented Parkinson's disease patients (PDD) were determined. Here, the mean values ± SD of the normalized concentrations (to the median of the PDND group) are shown. For avidity determination, the patient serum samples were untreated (Conc.) or treated with urea (Conc. + urea). *P*-values: ^a)^ PDND compared to PDD ^b)^ PDND: nAbs concentration compared to nAbs concentration + urea and ^c)^ PDD: nAbs concentration compared to nAbs concentration + urea.
(PDF)
