# Supplementary figures and images for: Naturally Occurring Autoantibodies against Tau Protein Are Reduced in Parkinson's Disease Dementia
Source: PLoS One. 2016 Nov 1;11(11):e0164953. doi: 10.1371/journal.pone.0164953 (PMC5089716; doi:10.1371/journal.pone.0164953)

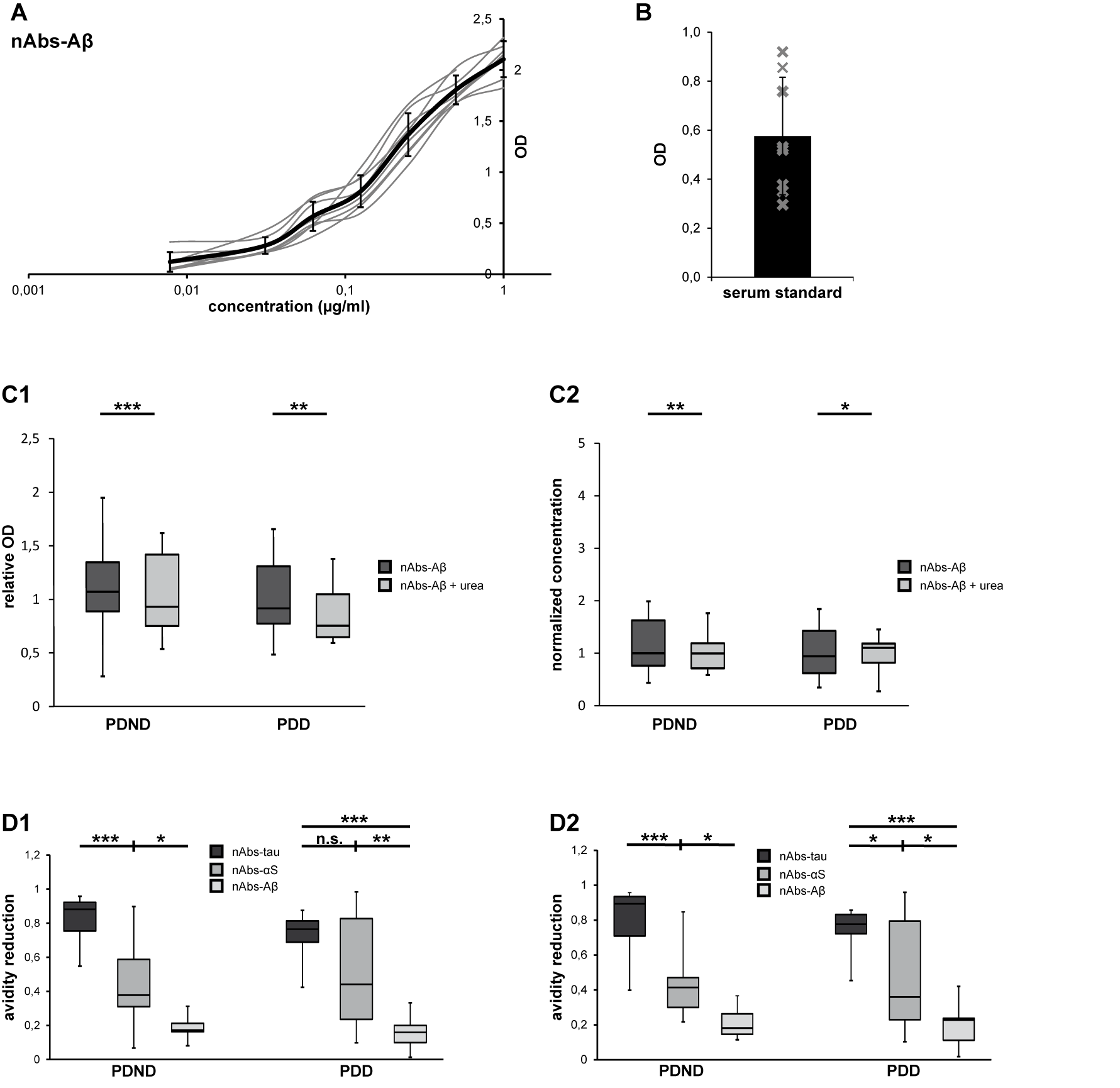

Supplement: S1 Fig — A+B. Considerable variation in the standard curves (A; grey: standard curves of single ELISA plates, black: mean standard curve) and a high interassay coefficient of variability (B; 41.7%; grey crosses: serum standard ODs of single ELISA plates, black: mean serum standard OD) demonstrate a low reproducibility. C: The relative serum sample OD (related to serum standard OD) of each non-demented (PDND) and demented Parkinson's disease (PDD) patient was determined without (nAbs) and with urea treatment (nAbs + urea). Based thereon and with the aid of the standard curves, nAbs-Aβ concentrations (normalized to the PDND group) were determined. D: With both evaluation methods, the urea mediated avidity reduction was determined. Both evaluation strategies are very similar and demonstrate no significant differences between PDND and PDD patients. However, within both patient groups, nAbs-Aβ are most avid, followed by nAbs-αS and nAbs-tau. Box plots show the median, 25% and 75% quartile. 50% of the generated data are located in the box and whiskers represent the minimum and maximum value. Corresponding mean values, standard deviations and p-values are outlined in S1, S3 and S4 Tables. (TIF) [file pone.0164953.s001.tif]

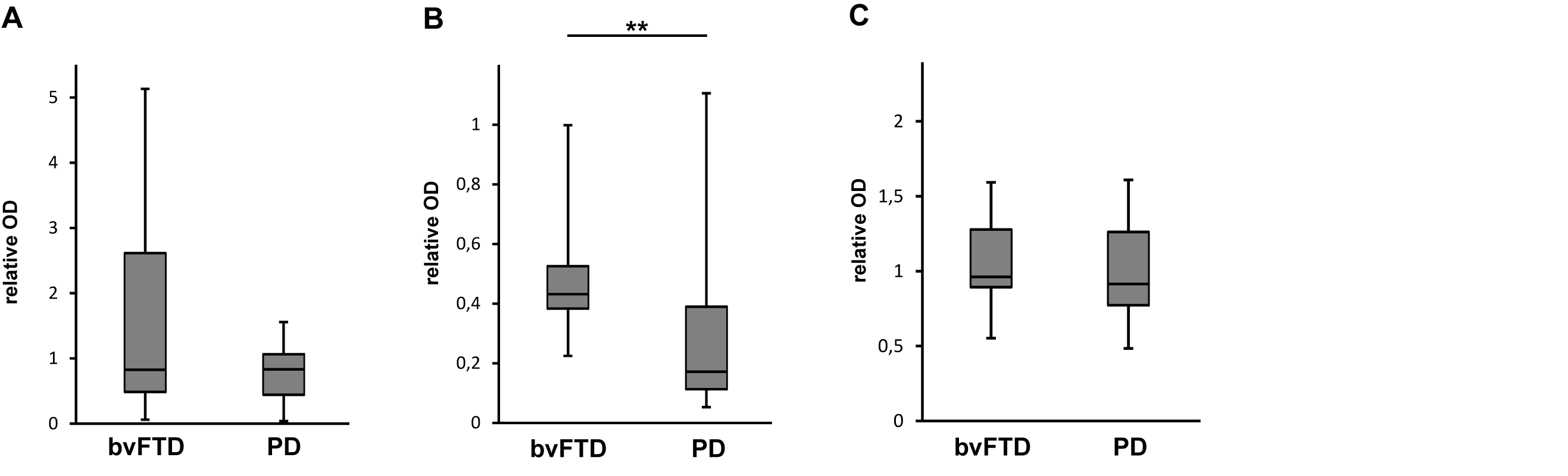

Supplement: S2 Fig — NAbs-tau- (A), nAbs-αS- (B) and nAbs-Aβ-ELISAs (C) were analyzed using the relative serum sample ODs (related to serum standard OD) of each Parkinson’s disease patient (PD, including PDND and PDD patients) as well as bvFTD patient. For an overview of the distribution of the serum sample ODs, box plots show the median, 25% and 75% quartile. 50% of the generated data are located in the box and whiskers represent the minimum and maximum value. FTD patients showed significantly increased nAbs-αS serum levels compared to PD patients (B; ***p < 0.001). (TIF) [file pone.0164953.s002.tif]

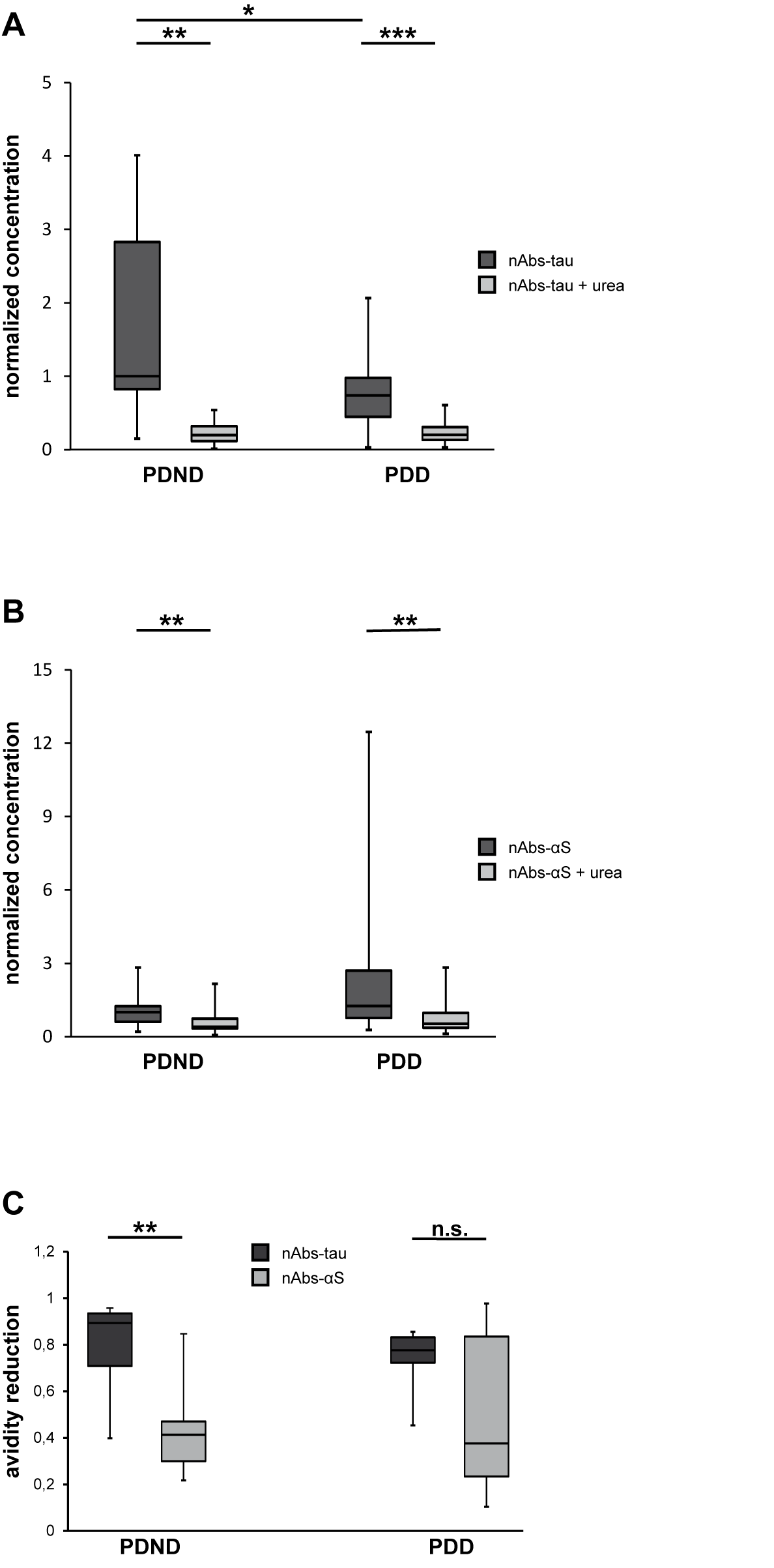

Supplement: S3 Fig — Using standard curves, the nAbs-tau and nAbs-αS serum concentration of each non demented (PDND) and demented Parkinson's disease (PDD) patient was determined. After normalization to the PDND group, nAbs-tau (A) and nAbs-αS (B) concentrations as well as the urea mediated avidity reductions (C) were compared between and within the two patient groups. Box plots show the median, 25% and 75% quartile. 50% of the generated data are located in the box and whiskers represent the minimum and maximum value. Corresponding mean values, standard deviations and p-values are presented in S3 and S4 Tables. (TIF) [file pone.0164953.s003.tif]
