# Supplementary material for: Naturally Occurring Autoantibodies against Tau Protein Are Reduced in Parkinson's Disease Dementia
Source: PLoS One. 2016 Nov 1;11(11):e0164953. doi: 10.1371/journal.pone.0164953 (PMC5089716; doi:10.1371/journal.pone.0164953)
Supplement: S1 Table — Relative serum sample ODs of non-demented (PDND) and demented Parkinson's disease patients (PDD) are shown as the mean ± SD for each ELISA. For avidity determination, the patient serum samples were untreated (OD) or treated with urea (OD + urea). P-values: a) PDND compared to PDD b)PDND: nAbs OD compared to nAbs OD + urea and c) PDD: nAbs OD compared to nAbs OD + urea. (PDF) [file pone.0164953.s004.pdf]

		PDND	*p*-value	PDD	*p*-value
**nAbs-tau**	OD	1.46 ± 0.93		0.75 ± 0.47	0.007[a)]
	OD + urea	0.22 ± 0.16	0.002[b)]	0.26 ± 0.18	<0.001[c)]
**nAbs-αS**	OD	0.15 ± 0.10		0.28 ± 0.28	0.053[a)]
	OD + urea	0.06 ± 0.05	0.001[b)]	0.10 ± 0.11	<0.001[c)]
**nAbs-Aβ**	OD	1.11 ± 0.48		1.00 ± 0.39	0.560[a)]
	OD + urea	1.04 ± 0.4	< 0.001[b)]	0.87 ± 0.31	0.009[c)]
