# Supplementary material for: Naturally Occurring Autoantibodies against Tau Protein Are Reduced in Parkinson's Disease Dementia
Source: PLoS One. 2016 Nov 1;11(11):e0164953. doi: 10.1371/journal.pone.0164953 (PMC5089716; doi:10.1371/journal.pone.0164953)
Supplement: S2 Table — Relative serum sample ODs of each Parkinson’s disease patient (PD, including PDND and PDD patients) as well as bvFTD patient are shown as the mean ± SD for each ELISA. For statistical analysis, Student's t-test or the Mann-Whitney-U test was applied. (PDF) [file pone.0164953.s005.pdf]

	bvFTD	PD	*p*-value
**nAbs-tau**	1.63 ± 1.46	1.09 ± 0.8	0.157
**nAbs-αS**	0.48 ± 0.19	0.22 ± 0.22	<0.001
**nAbs-Aβ**	0.97 ± 0.18	1.06 ± 0.44	0.333
