# Supplementary material for: Naturally Occurring Autoantibodies against Tau Protein Are Reduced in Parkinson's Disease Dementia
Source: PLoS One. 2016 Nov 1;11(11):e0164953. doi: 10.1371/journal.pone.0164953 (PMC5089716; doi:10.1371/journal.pone.0164953)
Supplement: S3 Table — Avidity reductions of the nAbs were calculated for each non-demented (PDND) and demented Parkinson's disease (PDD) patient as the quotient of the urea treated sample divided by the untreated sample. Therefore, the optical density (OD) as well as the concentration based evaluation method was used. Data are shown as the mean ± SD. P-values. a) PDND compared to PDD. b) nAbs-tau compared to nAbs-αS within patient groups (comparing two groups). c) nAbs-tau compared to nAbs-αS within patient groups (comparing three groups). d) nAbs-tau compared to nAbs-Aβ within patient groups (comparing three groups). e) nAbs-αS compared to nAbs-Aβ within patient groups (comparing three groups). (PDF) [file pone.0164953.s006.pdf]

	OD analysis			Concentration analysis		
	**PDND**	**PDD**	***p*-value[a)]**	**PDND**	**PDD**	***p*-value[a)]**
**nAbs-tau**	0.82 ± 0.14	0.73± 0.13	0.126	0.80 ± 0.18	0.75± 0.12	0.134
**nAbs-αS**	0.44 ± 0.26	0.52 ± 0.32	0.447	0.43 ± 0.19	0.48 ± 0.31	0.621
***p*-value[b)]**	< 0.001	0.057		0.003	0.074	
**nAbs-Aβ**	0,15 ± 0,08	0,15 ± 0,10	0.550	0,16 ± 0,09	0,18 ± 0,13	0.777
***p*-value[c)]**	< 0.001	0.1		< 0.001	0.027	
***p*-value[d)]**	< 0.001	< 0.001		< 0.001	< 0.001	
***p*-value[e)]**	0.022	0.009		0.022	0.039	
