# Supplementary material for: Naturally Occurring Autoantibodies against Tau Protein Are Reduced in Parkinson's Disease Dementia
Source: PLoS One. 2016 Nov 1;11(11):e0164953. doi: 10.1371/journal.pone.0164953 (PMC5089716; doi:10.1371/journal.pone.0164953)
Supplement: S4 Table — Using standard curves, nAbs serum concentrations of non-demented (PDND) and demented Parkinson's disease patients (PDD) were determined. Here, the mean values ± SD of the normalized concentrations (to the median of the PDND group) are shown. For avidity determination, the patient serum samples were untreated (Conc.) or treated with urea (Conc. + urea). P-values: a) PDND compared to PDD b) PDND: nAbs concentration compared to nAbs concentration + urea and c) PDD: nAbs concentration compared to nAbs concentration + urea. (PDF) [file pone.0164953.s007.pdf]

		PDND	*p*-value	PDD	*p*-value
**nAbs-tau**	Conc.	1.63 ± 1.29		0.76 ± 0.50	0.015[a)]
	Conc. + urea	0.22 ± 0.16	0.002[b)]	0.23 ± 0.17	<0.001[c)]
**nAbs-αS**	Conc.	1.14 ± 0.81		2.44 ± 3.01	0.091[a)]
	Conc. + urea	0.63 ± 0.60	0.001[b)]	0.83 ± 0.79	0.001[c)]
**nAbs-Aβ**	Conc.	1.15 ± 0.53		1.02 ± 0.56	0.460[a)]
	Conc. + urea	1.03 ± 0.40	0.004[b)]	0.98 ± 0.41	0.044[c)]
